# The Role of Avocados in Maternal Diets during the Periconceptional Period, Pregnancy, and Lactation

**DOI:** 10.3390/nu8050313

**Published:** 2016-05-21

**Authors:** Kevin B. Comerford, Keith T. Ayoob, Robert D. Murray, Stephanie A. Atkinson

**Affiliations:** 1 Department of Nutrition, University of California at Davis, Davis, CA 95616, USA; 2 Department of Pediatrics, Albert Einstein College of Medicine, Bronx, NY 10461, USA; keith.ayoob@einstein.yu.edu; 3 Department of Human Sciences, The Ohio State University, Columbus, OH 43210, USA; murrayMD@live.com; 4 Department of Pediatrics, McMaster University, Hamilton, ON L8S 4KI, Canada; satkins@mcmaster.ca

**Keywords:** avocado, monounsaturated fat, oleic acid, fiber, carotenoids, fetal health, maternal diet, pregnancy, lactation

## Abstract

Maternal nutrition plays a crucial role in influencing fertility, fetal development, birth outcomes, and breast milk composition. During the critical window of time from conception through the initiation of complementary feeding, the nutrition of the mother is the nutrition of the offspring—and a mother’s dietary choices can affect both the early health status and lifelong disease risk of the offspring. Most health expert recommendations and government-sponsored dietary guidelines agree that a healthy diet for children and adults (including those who are pregnant and/or lactating) should include an abundance of nutrient-rich foods such as fruits and vegetables. These foods should contain a variety of essential nutrients as well as other compounds that are associated with lower disease risk such as fiber and bioactives. However, the number and amounts of nutrients varies considerably among fruits and vegetables, and not all fruit and vegetable options are considered “nutrient-rich”. Avocados are unique among fruits and vegetables in that, by weight, they contain much higher amounts of the key nutrients folate and potassium, which are normally under-consumed in maternal diets. Avocados also contain higher amounts of several non-essential compounds, such as fiber, monounsaturated fats, and lipid-soluble antioxidants, which have all been linked to improvements in maternal health, birth outcomes and/or breast milk quality. The objective of this report is to review the evidence that avocados may be a unique nutrition source for pregnant and lactating women and, thus, should be considered for inclusion in future dietary recommendations for expecting and new mothers.

## 1. Introduction

The federal dietary recommendations in the U.S. only apply to Americans over the age of two years [1], yet one of the most critical times for proper nutrition is in the first two years of life when growth and development rates are at their peak. The development of federal dietary guidelines for maternal, infant, and toddler food patterns are due to be issued in 2020. Ideally, these guidelines should be based on foods and dietary patterns—not simply on nutrients—since the average American can understand and quantify food items much more accurately than individual nutrients. Furthermore, the federal recommendations for pregnant and lactating mothers, and for infants and toddlers, may be more practical and applicable if they included specific food items that are rich in multiple shortfall nutrients and low in empty calories. Doing so would help caregivers better understand what foods in each food group are actually recommended, instead of needing to interpret more complicated nutrient-based recommendations.

The basic cornerstones of a healthy diet for children and adults should include nutrient-rich foods such as fruits and vegetables, which contain a variety of essential nutrients and other health-promoting non-essential compounds such as fiber [2]. These foods should ideally be rich in shortfall nutrients identified in the 2015–2020 Dietary Guidelines for Americans (DGA)—calcium, vitamin D, potassium, and fiber [1]—as well as in key essential nutrients such as folate and iron, which have garnered global scientific support from the World Health Organization and the Food and Agriculture Organization (WHO/FAO) for their beneficial effects on mother-offspring health outcomes [1,3]. For example, breast milk is the ideal food for newborns and infants since it is rich in both essential and shortfall nutrients that are necessary for proper health and development, along with several bioactive compounds which can potentially modulate facets of immunity, digestion and nutrient uptake. Similarly, unsaturated oil-containing fruits such as avocados provide multiple shortfall nutrients without significantly contributing to any of the 2015 DGA nutrients of concern for overconsumption (*i.e.*, sodium and saturated fat), or to empty calories from added sugars (Table 1). Furthermore, the 2015 Dietary Guidelines for Americans Committee (DGAC) report indicates that several other vitamins found in avocados (*i.e.*, vitamin E, folate, and vitamin C) are currently under consumed relative to the estimated average requirement (EAR) for Americans above two years old [2]. Although these nutrients of concern have not yet been directly studied by the DGAC for infant/toddler populations, they are required in higher amounts by pregnant and lactating women compared to the general population [4]. In addition to containing multiple shortfall nutrients, avocados are a source of several promising non-essential compounds, such as monounsaturated fats (MUFA), lipid-soluble antioxidants, and various phytosterols that show promise for maternal, infant, and toddler health (Table 1).

Proper nutrition is never more critical for insuring the quality of human health and reducing the risk for disease than for mothers in the perinatal and neonatal periods, and for infants and toddlers in their first years of life [5]. However, these are difficult populations in whom to conduct clinical experiments due to ethical constraints. There is a need for review of dietary components that may influence health and development during these life stages. These concerns are being addressed by the Birth to 24 months and Pregnant Women Dietary Guidance Development Project expert work groups convened by the U.S. Department of Agriculture (USDA) and Department of Health and Human Services (HHS) [6].

Our paper, the first of a two-part series, covers a large body of epidemiological evidence, and a much smaller body of clinical research that has investigated the effects of dietary patterns, dietary components, and individual nutrients on maternal nutrition during the critical periods of conception, gestation, and lactation. The objective of this report is to review the evidence that dietary patterns which include avocados may provide both nutritive and bioactive components that are ideal for pregnant and lactating women, and thus additional research into the role of avocados should be performed in these populations. Throughout, where applicable, the paper addresses nutrition topic questions posed by the Birth to 24 months and Pregnant Women Dietary Guidance Development Project expert work groups. Two of the four areas of focus for the development project’s working groups—Work Group 1—Infancy: Period of Sole Nutrient Source Feeding (0–6 months), and Work Group 4—Caregivers (Mothers and Others)—Factors Influencing Nutrient Needs, Infant Feeding Choice, Dietary Quality and Food Habits—are to investigate factors influencing maternal nutrient needs and infant nutrition extending from pregnancy until the first foods are introduced. While these sub-groups have each been tasked with answering dozens of questions on their respective topics, this report focuses on the following questions that are most closely related to food (especially fruits and vegetables) and nutrient intake, and their effects on birth outcomes and health outcomes:

- What is the influence of maternal dietary intake on micronutrients—including fat-soluble and water-soluble vitamins—and macronutrients—including total fat, *n*-3 polyunsaturated fatty acids (PUFA), *n*-6 PUFA, and *trans* fats—on human milk composition?
- What are the effects of dietary patterns—such as vegan, vegetarian, macrobiotic diets—on breast milk composition?
- What is the relationship between maternal dietary water-soluble vitamin intake and human milk water-soluble vitamin composition?
- What is the relationship between maternal dietary-fat intake and human milk-fat composition?
- What is the relationship between maternal dietary fat-soluble vitamin intake and human milk fat-soluble vitamin composition?

## 2. General Recommendations for Maternal Diet during the Preconceptional Period, Pregnancy, and Lactation

Maternal nutrient intake can affect every major aspect of reproduction from the early peri-conceptional period to the later post-natal stages. In essence, maternal nutrient status influences the entire range of maternal functions: the ability to conceive and maintain a healthy pregnancy [8], produce an effective placenta, assist the offspring’s brain and body development, and manufacture adequate and nutritious breast milk [9]. Additionally, the maternal host environment during pregnancy influences gene expression and the health of the offspring for years after birth [10]. It remains unclear just how long before conception the importance of maternal nutrition—and even paternal nutrition [11]—is for the short-term and long-term health of the offspring. What is known is that proper maternal nutrition—especially for key nutrients and bioactives found in fruits and vegetables—is paramount for reducing the risk for congenital birth defects in the critical periods directly before and after conception occurs [12]. Optimal nutrition is a critical factor among all women of childbearing age, even before conception; however, many young mothers are not getting the foods and nutrients they need for themselves or their offspring [13].

The nutrients most commonly associated with prenatal and neonatal health are: iodine, iron, zinc, vitamin A and carotenoids, vitamin D, choline, folate, riboflavin, vitamin B-6 and vitamin B-12, protein, and several specific fatty acids [14,15,16]. Yet, pregnant women in the U.S. are known to have intakes of folate, potassium, fiber, and vitamins A, D, E, and C well below the EAR [2]. Thus, major deficits can occur especially for nutrients like iron and vitamin B6 which are required in nearly twice the normal recommended dietary allowance of the mother to promote a proper host environment and/or used to nourish the offspring by being passed along through the placenta or breast milk. The addition of healthy and nutrient-dense foods are ideal options for assisting expecting and lactating mothers in reaching their nutritional goals.

## 3. Adjusting and Improving the Federal Dietary Advice for Pregnancy and Lactation

Failure of women to meet the recommended guidelines during the perinatal period is well documented [17,18,19,20]. Whether this is due to unawareness, inability to adhere to, or simply an ambivalence towards the federal dietary recommendations is not clear. What is apparent is that the effects of suboptimal dietary choices in U.S. women are associated with increasing rates of maternal obesity [21] and gestational diabetes [22], both of which increase risks for birth defects [23] and affect lactation [24]. In this regard, the DGA may be too abstract for many expecting American mothers. For example, the federally run website ChooseMyPlate.gov specifically recommends fruits and vegetables that provide potassium and provitamin A for pregnant and breastfeeding mothers [25]. This is ambiguous advice, however, since many Americans do not know which fruits and vegetables best contain these nutrients and why they are important. This lack of understanding tends to lead to suboptimal dietary patterns and an unnecessary deference to supplements instead of food as a primary means of meeting their—and their offspring’s—nutritional needs during pregnancy and lactation [26,27]. In order to address nutrition in a way that is more consumer-friendly and impactful, further efforts should be undertaken by the DGA to simplify the dietary recommendations for pregnant and lactating women. For example, providing examples of specific food items—not just nutrients or broad food groups—that are rich in multiple recommended nutrients—such as “eat more salmon, yogurt, walnuts or avocados”—will undoubtedly be better understood by those trying to figure out what to eat.

Currently, the federal dietary advice in the U.S. for pregnant mothers is largely based on what not to eat, such as recommendations to avoid alcohol and empty calories from added sugars and saturated fats [28]. This is valuable advice, but could be improved upon if it gave real-world examples on how to exchange a nutrient-poor food for a nutrient-rich one without losing flavor or textural properties. Recent research from the French Nutrition and Health Survey shows that food substitutions of this nature are a promising and effective dietary strategy for improving nutrient adequacy in the diet of adults [29]. For example, a simple substitution recommendation could be to choose fresh avocado over mayonnaise on a sandwich to reduce saturated fats while adding numerous other essential nutrients, potentially bioactive compounds (e.g., lipophilic antioxidants and phytosterols), and fiber. Another example would be to use an avocado- and yogurt-based dressing in place of many nutrient-poor commercial options in order to avoid added sugars and saturated fats while adding protein, fiber, and fat-soluble vitamins.

## 4. Maternal Diet: Effects on Fertility, Fetal Growth, and Birth Outcomes

Pregnant women have a higher requirement for many essential and non-essential nutrients during gestation. The most heavily researched nutrients for fetal health can be narrowed down to a few different groups: (1) micronutrients that regulate DNA synthesis, cell division, and growth (*i.e.*, folate, B-12, vitamin A, vitamin D, iron, and zinc); (2) nutrients that assist with brain development (*i.e.*, iodine and specific fatty acids); and (3) antioxidant nutrients which protect against free radical damage and DNA mutation (*i.e.*, vitamin A and carotenoids, vitamin C, and vitamin E). Another important class of nutrients for fetal health not currently recognized is regulatory nutrients—such as fiber and potassium—which may improve maternal health status (*i.e.*, reduce the risk of diseases such as hypertension, dyslipidemia, and gestational diabetes) [30,31,32], thereby potentially producing a more favorable host environment and reducing pregnancy and birth complications associated with maternal disease [31,33].

Infertility affects over 10% of U.S. women of reproductive age [34], or approximately one in six couples during their reproductive years [35]. While the current state of scientific knowledge on preconception diets is improving, there is still limited information available to date [11]. Studies that have investigated the effects of diet on fertility have shown that weight loss in overweight and obese women can improve insulin sensitivity, which is a key factor in improving fertility [8,36,37]. Studies have also shown that certain foods or diet plans can improve fertility [38]—in particular, diet plans involving increased fruit and vegetable intake, as well as increased intake of certain high-fat foods (e.g., dairy foods, foods like avocados that contain unsaturated plant oils, and fish oils) and reduced intake of other high-saturated fat foods (*i.e.*, red and processed meat, and foods that contain *trans* fats) [8].

### 4.1. Mediterranean-Style Diet and Fertility

The 2015 DGAC report recognized several dietary patterns (e.g., USDA-style, vegetarian-style, and Mediterranean-style) that can support beneficial health outcomes for the general population, including pregnant and breastfeeding mothers [2]. A particularly well-researched eating plan associated with general health and maternal health is a Mediterranean-style diet. A Mediterranean-style diet varies by region, but is traditionally based on regular intake of antioxidant- and fiber-rich fruits and vegetables, lean choices of protein, omega-3s in the form of fatty fish, whole grains, and MUFA from plant oils. A maternal Mediterranean-style diet has been associated with significantly improved health outcomes such as lower total and low-density cholesterol levels for the mother and up to a 90% lower risk for preterm delivery [39].

While avocados are not part of the “traditional” Mediterranean-style diet, according to the Mediterranean diet pyramid created by Oldways (a non-profit food and nutrition education organization), along with the Harvard School of Public Health and the WHO, avocados are Mediterranean-style foods because they are classified as an antioxidant- and fiber-rich fruit and have a fatty acid profile that is naturally rich in MUFA [40]. Two-thirds of the fatty acid content of avocados are MUFA. Several recent studies have shown that a greater adherence to a Mediterranean-style diet may enhance fertility rates by reducing the risk of obesity, hypertension [41], insulin resistance [42], and diabetes [43] in pregnant women. A Mediterranean-style diet has been associated with nearly a 70% lower risk of ovulatory disorders in infertile women [8] when compared to diets that are high in *trans* fats.

### 4.2. Low-Glycemic Diets: Effects on Fertility, Maternal Health, and Fetal Health Outcomes

**Figure 1:**
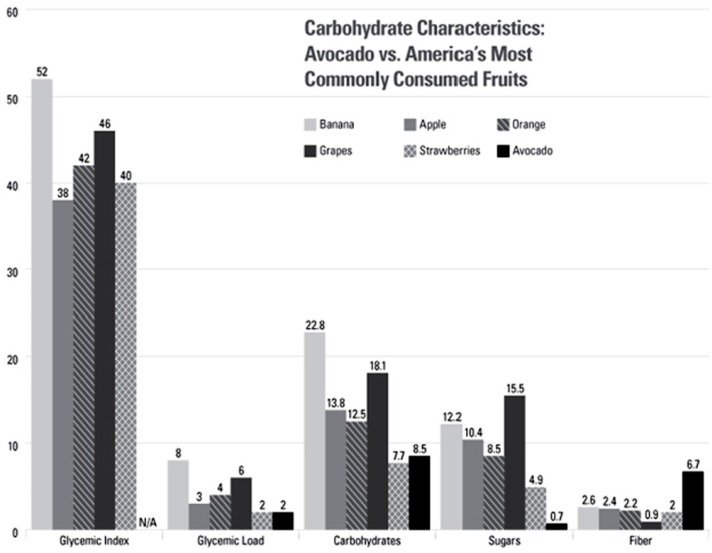
Carbohydrate Characteristics: Avocado vs. America’s Most Commonly Consumed Fruits. Glycemic Index Scale: Glucose = 100. Carbohydrates, sugars, and fiber are all listed per 100 g serving. There are no glycemic index values given for avocados because they contain so few carbohydrates that it would be difficult for people to consume a large enough portion (50 g of available carbohydrates) to properly perform glycemic index testing. Data sources: Nutrients—USDA Nutrient Database for Standard Reference 27 [47,48]; Glycemic Load and Glycemic Index—International table of glycemic index and glycemic load values [49].

The association between a low-glycemic maternal diet and birth outcomes begins in the pre-pregnancy period, with a lower intake of high-glycemic foods having been shown to increase the chances of fertility [44,45]. An analysis of the Nurses’ Health Study population showed that higher intakes of MUFA, vegetable protein, fiber, and low-glycemic carbohydrates were associated with improved fertility outcomes in the Nurses’ Health Study II population [35]. All of the dietary components listed above are also dietary components (or tenets) of a Mediterranean-style diet [46] and oil-containing fruits such as avocados (Figure 1). It should be noted that this research was gathered from women with no history of infertility [35] so it is still unknown how these findings apply to women with known fertility issues.

Randomized trials show that a low-glycemic index diet can be used in the management of gestational diabetes by reducing an expectant mother’s need for insulin [50] and by improving maternal glycemia [51]—all while reducing the negative effects of maternal insulin resistance and hyperglycemia on the developing fetus. When compared to a high-glycemic diet, the effects of a low-glycemic diet are further seen (or more importantly not seen) throughout the offspring’s early life in the form of a reduced rate for birth defects (*i.e.*, encephalocele, diaphragmatic hernia, small intestinal atresia/stenosis, and atrial septal defects) [23]. A low-glycemic maternal diet has also been associated with offspring birth weight, birth length, adiposity, and arterial wall thickness [52,53], and later life reductions in biomarkers for metabolic syndrome (*i.e.*, insulin levels, leptin levels, and homeostatic model assessment of insulin resistance [HOMA-IR]) [54]. Overall, dietary patterns that are based on nutrient-rich, low-glycemic foods, such as legumes, non-starchy vegetables, and oil-containing fruits, offer great building blocks for a nutritious dietary pattern for both a mother and her offspring.

### 4.3. Maternal Intake of Fruits and Vegetables

Fruits and vegetables are nutrient-dense foods and key components of all USDA recommended dietary patterns. Fruits and vegetables have been linked to reductions in numerous types of disease and deficiency states [55,56], largely because they contain essential nutrients and bioactive compounds [57], but also because consumption of fruits and vegetables generally displaces the consumption of other less nutrient-rich foods. Many of the essential nutrients in fruits and vegetables are the same nutrients that are recommended for mothers during the periconceptional and perinatal periods. [58]. Fruits and vegetables are also whole-food sources of fiber, magnesium, and vitamin C, as well as thousands of relatively unstudied bioactive compounds, including various antioxidants and phytosterols [59,60], which may also have modulatory effects on pregnancy outcomes. At present, the available evidence regarding maternal consumption of fruits and vegetables, especially those rich in multiple short-fall nutrients, supports their importance as part of a healthy and protective diet throughout pregnancy and early life [58,61,62]. In both highly developed and developing countries, however, fruit and vegetable intake levels for pregnant women are typically much lower than recommended and may contribute to increased risk of poor fetal development [58].

After conception occurs, fetal growth rate is one of the most useful indicators of an offspring’s ability to survive and thrive later in life. Approximately 8% of infants in the U.S. are born with a low-birth-weight [63]. A suboptimal fetal growth rate—as well as an excessive fetal growth rate—has been associated with developmental issues in early life, childhood, and adolescence. Additional evidence even suggests that poor fetal nutrition increases the risk for poor health outcomes such as obesity, impaired bone health, immune dysfunction, impaired mental health, and cardiometabolic disease (e.g., cardiovascular disease and type 2 diabetes) many years later in life [63,64,65].

The growth and development of a fetus is dependent on several factors, some of which may occur before conception. Some data suggest fetal growth and development can be influenced by the nutritional status of the mother prior to and at the time of conception [66], but these findings have yet to be confirmed. What is currently known from epidemiological studies is that nutrient-poor maternal nutrition is associated with fetal disease [67] and below average fetal growth [63]. In regards to fetal health, the data suggest that an imbalance between dietary intake of macronutrients and micronutrients contributes to the pathogenesis of complex birth defects, and that specific dietary modifications, such as increased consumption of fruits and vegetables, may be able to help reduce the risk and severity of various defects.

In 2004, a case-control study of 206 mothers with a child that had a non-syndromic orofacial cleft and 203 control mother-child dyads, showed that a higher pre-conceptional intake of nutrients predominantly present in fruits and vegetables (*i.e.*, fiber, vegetable protein, beta-carotene, ascorbic acid, alpha-tocopherol, magnesium, and iron) was associated with a lowered risk for orofacial clefts in the offspring [68]. However, this study was limited in that all of the participants were Caucasian, and confounders such as body mass index (BMI) and physical activity were not adjusted for. Additionally, in 2004, a similar case-control study involving Caucasian women investigated the associations between maternal diet and risk for spina bifida in the offspring (106 cases and 181 controls). The researchers found that low pre-conceptional intakes of plant-based nutrients were associated with a two- to five-fold increase in spina bifida risk [69]. Taken together, these findings strengthen the advice that in order to lower the risk for orofacial and neural-tube defects in their children, women of child-bearing age should consume a balanced diet, with nutrient-rich fruits and vegetables before and after conception.

Fruit and vegetable intake is also associated with a reduced risk for preeclampsia (*i.e.*, maternal hypertension and proteinuria) [33,70] and insulin resistance [71]. When compared to the relatively low-calorie and low-protein options provided by most fruits and vegetables, it seems reasonable that birth weight is most strongly correlated with high-protein and high-calorie foods since those foods are associated with muscle and adipose tissue expansion in adults. Surprisingly, a 2006 prospective cohort study of 44,612 Danish women found that when pregnant mothers followed a high-calorie, high-protein Western-style diet (*i.e.*, higher amounts of red and processed meat, low fruit and vegetable intake, *etc*.), they tended to have an increased risk for low birth weight for gestational age compared to those who consumed fewer calories and greater quantities of plant foods [64]. A much smaller study of 2466 rural Indian mothers and their babies also showed similar results, where higher intakes of protein and calories by the mother were not associated with offspring birth weight, but green leafy vegetables and fruits were [72]. Therefore, it is unclear as to whether certain components of a high animal-protein and high-fat diet impair fetal growth, or if certain components in fruits and vegetables—such as key micronutrients—are uniquely responsible for proper fetal growth. It is most likely not simply one or the other, but rather that animal-based foods, fruits, and vegetables can all modulate fetal growth to varying degrees. The association found between fruit and vegetable intake and birth weight suggest the existence of potential micronutrient or phytochemical combinations present in plant-based foods that play an important role in optimal fetal development [62]. The types of nutrients from fruits and vegetables and their roles in fetal development deserve further investigation.

## 5. Maternal Intake of Key Avocado Compounds: Effects on Fertility, Fetal Health, and Birth Outcomes

### 5.1. MUFA—Oleic Acid

Among the classes of nutrients most frequently associated with fertility are lipids—especially fatty acids and fatty acid ratios—which appear to be key modulators of human fecundity [73]. The majority of research on fatty acid intake in pregnant mothers has focused on essential fatty acid intake—especially long-chain polyunsaturated fatty acids (LCPUFA) such as DHA. MUFA, however, also deserve attention for fetal health and birth outcomes.

The association between intake of MUFA in pregnant women and their offspring was demonstrated by Agostoni *et al.* who collected blood on 16 healthy women and their newborns to determine their whole blood-fatty acid profile. They found that MUFA made up approximately 29% of the blood fatty acids of pregnant mothers, 18% of the umbilical cord blood, and 23% of the blood of a newborn infant [74]. The MUFA oleic acid (18:1*n*-9) comprised approximately 75%–85% of the total MUFA in these blood compartments. Additional research by Agostoni *et al.* on 144 infants showed that MUFA levels were significantly lower in newborn infants who were small for gestational age when compared to those who were born appropriate for gestational age (23% *vs.* 25%, respectively) [75]. There were no differences in total PUFA or saturated fatty acids (SFA) in blood lipid profiles between groups. It is unclear from the report as to why this difference occurred, or as to whether increased MUFA consumption by the mother was responsible for the difference in size. Nonetheless, it is clear that MUFA (especially oleic acid) make up a large portion of an infant’s blood fatty acid profile, and that the role of MUFA in gestational development should continue to be investigated. Furthermore, a recent case-control study of 11 cases of gastroschisis (a congenital birth defect where the baby’s intestines and other internal organs can push out through a hole in the abdominal wall) and 34 controls, provided evidence that a peri-conceptional maternal diet rich in oleic acid may be able to significantly lower the odds of gastroschisis in the offspring [76]. In addition, mothers who had higher intake of vegetables had an even lower chance of having an infant with gastroschisis.

In their analysis of the Nurses’ Health Study II, Chavarro and Willet analyzed the effects of lipid intake (cholesterol, fatty acids, and fatty acid ratios) on fertility in more than 18,500 women in the U.S. The researchers found that consuming just 2% of energy from unprocessed MUFA instead of hydrogenated *trans* fats was associated with less than half of the risk of ovulatory infertility [8]. A potential explanation for these findings is that certain unsaturated fatty acids, such as unprocessed MUFA from fruits or vegetables, can bind to the peroxisome proliferator-activated receptor γ (PPAR-γ), and thereby reduce inflammation and improve ovulatory function [77]. The researchers concluded that higher intake of unprocessed MUFA (commonly found in non-hydrogenated vegetable oils and oil-containing fruits) and lower intakes of *trans* fats may lead to lower incidences of ovulatory infertility [8]. A further study by Chavarro *et al.* investigated the effects of fat intake on preclinical and clinical outcomes in women undergoing *in vitro* fertilization (IVF) [78]. The researchers found that greater intakes of MUFA were related to nearly three and half times higher odds of live birth after embryo transfer, compared to lower intakes of MUFA. A recent critical review of the available literature on diet and fertility by Sinska *et al.* came to similar conclusions as Chavarro and Willet. After reviewing the current evidence, the Sinska group suggested that a larger intake of MUFA can help to improve a woman’s fertility, while the intake of *trans* fats should be avoided [44]. Avocados are a well-tolerated food that can serve as an important source of lipids such as MUFA. An ounce of avocado provides 4.6 g total fat, 3 g of that is MUFA—primarily in the form of oleic acid [79].

### 5.2. Fiber

Fiber intake by Americans is low enough to be of public health concern [1]. According to the 2015 DGAC report, only 8% of women who were pregnant had adequate intake of fiber [2]. In 2012, Blumfield *et al.* performed a review of dietary intakes of pregnant women in developed countries and found that fiber intakes were consistently below the recommended levels [80]. Although fiber is generally not considered an essential nutrient—sometimes it is even referred to as an “anti-nutrient” since it can inhibit the absorption of certain nutrients [81]—it is still an important dietary component for maternal and fetal health [9,61]. Low fiber intake is associated with an increased risk for several maternal diseases (*i.e.*, chronic constipation, type 2 diabetes, and hypertension/preeclampsia) that can all dramatically affect the fetal environment [9,82]. Studies on fiber intake in pregnant women and the risk for preeclampsia and gestational diabetes consistently encourage greater maternal fiber intake for a reduced risk for both diseases [31,33,83].

Although there are different types and forms of dietary fiber, most of the observational research on fiber intake during pregnancy does not distinguish which type of fiber was consumed, and usually just aggregates all fibers into a general intake value. Many whole plant foods contain either predominantly soluble or insoluble fiber, but avocados contain a mix of both. Even a modest portion of avocado—30 g—contains 2 g of fiber, with a ratio of 70% insoluble to 30% soluble fiber [84]. Higher fiber intake in pregnant mothers has been shown to attenuate pregnancy-associated dyslipidemia, which along with hypertension, is an important clinical characteristic of preeclampsia [31]. Soluble and insoluble fiber intakes have both been shown to be associated with a lower relative risk for preeclampsia (soluble: RR = 0.30; 95% CI = 0.11–0.86 *vs.* insoluble: RR = 0.35; 95% CI = 0.14–0.87) [31]. Any compound that can reduce the risk for maternal disease during pregnancy should, in theory, also be beneficial for the developing fetus, which is heavily influenced by the mother’s nutrient status and disease status.

### 5.3. Folate

Approximately 3% of U.S. babies are born with a birth defect [63]. The best known nutrient for reducing the risk for birth defects (*i.e.*, neural tube defects and some heart defects) is folate/folic acid. Folate is a cofactor for many essential cellular reactions, including DNA and nucleic acid synthesis. During pregnancy, the folate requirement for a mother increases due to new cell and tissue formation (*i.e.*, increase in red blood cell mass, enlargement of the uterus, development of the placenta, and growth of the fetus), and it is recommended that pregnant women consume 600 μg of dietary folate equivalents daily from all food sources [85]. Insufficient maternal folate intake has been linked to increased rates of low birth weight, preterm birth, cardiac defects and neural tube defects [14,15,86,87]; however, the risk for all of these outcomes can be significantly reduced with adequate intake. In addition, supplements and fortified foods, the highest food sources of folate tend to be beans, leafy green, and cruciferous vegetables. Avocados are also a source of this nutrient. A 30 g serving of avocado contains approximately 27 μg of folate [79], which is higher than a serving of most fruits, tree nuts, and seeds [79,88]. Based on a recent National Health and Nutrition Examination Survey (NHANES) survey, the average daily avocado consumption by persons who eat avocados is over twice that amount (70 g), at approximately one-half of a medium sized avocado [89]. At this level of average intake, avocado is a good source of folate providing approximately 62 μg (*i.e.*, 10%) of the recommended daily intake of folate per day for pregnant women.

### 5.4. Vitamin A and Carotenoids

Vitamin A and carotenoids are needed for proper health throughout a person’s entire life span, but their effects are most critical during life stages when cells are rapidly proliferating and differentiating, such as during fetal development and infancy [90]. Some carotenoids have vitamin A activity (e.g., beta-carotene, alpha-carotene and beta-cryptoxanthin) while others do not (e.g., lutein, lycopene, and zeaxanthin). All of these compounds have antioxidant properties, and they exhibit a range of functions involving eye health, immune function, and neurological development [90]. A deficiency in vitamin A, especially at critical times in development, can cause a host of severe health issues such as blindness and immunodeficiency complications [91], whereas intakes of various carotenoids, whether they have vitamin A activity or not, are associated with immune health [92,93], eye health, and brain development [94].

Henriksen *et al.* have suggested that levels of carotenoids are depleted through placental transfer to the fetus during pregnancy, thereby the dietary need increases for carotenoid intake by pregnant mothers in order to avoid deficiencies in both the mother and the offspring [95]. Lutein and zeaxanthin are critical for proper eye development *in utero*, making them key carotenoids for fetal development especially in the third trimester [95]. These particular carotenoids are either not present in, or not well absorbed from, most fruits and vegetables; however, they are both present in avocados and well-absorbed due to the fatty acid content of avocado. For example, carotenoid absorption has been shown to be improved by 5–15 times when avocado is present in a salad, when compared to an avocado free salad [96]. The fetal demand for these nutrients suggest that maternal intake of carotenoids should be monitored closely, with emphasis on beta-carotene, lutein, and zeaxanthin [95]; and adequate dietary fat to encourage their absorption. The fatty acid and fat-soluble carotenoid (especially lutein and zeaxanthin) composition of avocados make them an ideal food for assisting pregnant mothers in attaining the nutrients necessary for proper early brain, eye, and immune development of their offspring. One ounce of avocado contains approximately 3.2 RE (20 μg) of beta-carotene, and 80 μg of lutein + zeaxanthin [79], which is absorbed in considerably higher quantities than a serving of most fruits and vegetables [97].

### 5.5. Potassium

Potassium is considered a shortfall nutrient by the 2015 DGA report [1], thus intakes are low enough to be a public health concern. On one hand, according to the What We Eat in America (WWEIA) data from 2007 to 2010, the usual intake distributions for pregnant and non-pregnant women in the U.S. ages 19–50 years show that only 3% of women had intakes above the adequate intakes (AI) for potassium [2]. On the other hand, most Americans tend to get too much sodium from their diets. An imbalance between high levels of dietary sodium and low levels of dietary potassium is associated with hypertension; and hypertension can increase the risk for stroke, cardiovascular disease, and insulin resistance [98,99]. Pregnant woman without a history of hypertension are only at a 3%–5% higher risk for developing preeclampsia, while 17%–25% of women with chronic hypertension will develop preeclampsia [100]. Both hypertension and preeclampsia can be harmful to fetal health, resulting in higher congenital malformations, particularly cardiac defects [101]. Higher potassium intake than sodium intake has been shown to blunt the effects of sodium on blood pressure in populations with hypertension [102], further supporting the importance of obtaining adequate dietary potassium for maternal health.

The influence of dietary potassium on blood pressure was demonstrated by, Kazemian *et al.* who performed a study on women with gestational hypertension that showed the odds of getting gestational hypertension decreased significantly with roughly 250–300 mg higher intakes of potassium per day [103]. This is less than the amount of potassium found in one-half of a medium-sized avocado, but more than in one-half of a medium sized banana or a large apple. Frederick *et al.* independently arrived at similar conclusions when investigating the effects of diet on preeclampsia [33]. Their results showed diets high in potassium (>4.1 g/day) are associated with an odds ratio of 0.49 for preeclampsia compared to lower potassium diets (<2.4 g/day). Pregnant mothers and their offspring can likely benefit from increasing their potassium intake while maintaining or lowering their sodium intake. A multicenter longitudinal study on teenage girls has also shown that even when sodium intakes are above recommended levels, higher potassium intakes were associated with lower systolic and diastolic blood pressure [104]. Avocados have more potassium by weight than most other common fruits and vegetables [105]; they contain roughly 152 mg potassium per ounce, and only 2 mg sodium [79], which can help pregnant women meet recommendations.

## 6. Maternal Intake of Fruits, Vegetables, and Key Avocado Compounds: Effects on Milk Production and Composition

Breast milk is recommended as the only necessary source of nutrition for the first months of life [106]; and human milk is often used as the basis from which to derive nutrient requirements for infants during the first year of life. While the influence of fruit and vegetable intake on breast milk production and composition is unknown, maternal consumption of fruits and vegetables is associated with specific flavor preferences in breastfed infants from the variety of food flavors received through the milk [107,108]. These early flavor experiences may help explain why infants who are breastfed tend to be more willing to try new foods, which may contribute to greater fruit and vegetable consumption later in life [109,110,111].

Maternal intake of some but not all nutrients can be reflected in the nutrient composition of breast milk [112,113]. For example, maternal folate intake does not significantly alter breast milk folate despite being a proven critical nutrient for fetal development [113]. In contrast, maternal intake of vitamin A, vitamin B6, and vitamin B12), as well as iodine and fatty acids directly influence the composition of breast milk [112,113,114]. Few fruits or vegetables are rich in both vitamins and fatty acids, with the exception of oil-containing fruits such as avocados, which contain MUFA.

### 6.1. MUFA—Oleic Acid

Human breast milk provides more than 50% of its energy from fat. The fatty acids in breast milk can be taken up directly from circulation (*i.e.*, from food or from mobilized fat stores) into the mammary glands and used in milk production, or they can be synthesized in the liver or mammary glands as needed [115]. Breast milk supplies SFA, PUFA, and MUFA [116], and the specific fatty acid profile is heavily implicated in infant health and development [112]. The primary fatty acids in breast milk are palmitic acid (16:0), linoleic acid (18:2*n*-6), and oleic acid (18:1*n*-9). These three fatty acids account for roughly three-fourths of the fatty acids in human milk [117]; with the MUFA oleic acid being the most abundant fatty acid of the three. Oleic acid is also the same fatty acid that is most abundant in oil-containing fruits such as avocados and olives, but the sodium content of ready-to-eat olives, ranging up to 1000 mg per serving, make them less than ideal as a daily food for pregnant and lactating mothers.

The quantity of total fat in the milk is fairly stable even with changes to the maternal diet [118]; however, the ratio of specific fatty acids in breast milk show extreme sensitivity to maternal nutrition. Several researchers have shown that the mother’s dietary habits can impact specific short-, medium, -and long-chain fatty acids in their milk, but the SFA content of the milk is generally unaffected by maternal dietary patterns [116,119]. Early researchers discovered that maternal dietary fatty acids are rapidly transferred into breast milk and within a few days the quality of human milk fat can be significantly influenced by dietary fat [120]. Therefore, the consumption of higher quantities of unsaturated fatty acids such as oleic acid by a nursing mother can be used to increase the oleic acid ratio in her milk, while a higher maternal intake of SFA does not get passed along in the breast milk in the same manner—possibly as a protection mechanism to maintain the fluidity of the breast milk, and/or to protect the infant from high levels of SFA in its early developmental stages.

Maternal lipid intake is the single most influential factor contributing to breast milk fatty acid composition [112], while carbohydrate intake has lesser influence [106]. A study in Brazilian mothers who consumed fruits four to six times per week, and whose primary fat source was soybean oil, showed that approximately 40%–42% of the fat calories in their milk came from SFA and another 20%–30% from PUFA [116]. On one hand, these values did not change much with variations in what the mother consumed. On the other hand, approximately 30%–35% of the fat calories in their milk came from MUFA, and this value was shown to change significantly depending on eating habits. Studies on European and Israeli mothers have shown that MUFA levels in their breast milk account for roughly 30%–45% of the total fatty acids, with oleic acid accounting for over 90% of the MUFA [117,121]. Furthermore, studies on Western women show that they have several-fold more *trans* oleic acid isomers in their milk than their non-Western counterparts because they ingest a higher percentage of partially hydrogenated oils in their diets [122]. The *trans* oleic acid isomers can be transferred to the fetus via cord blood and to the infant through milk. These *trans* isomers may have deleterious effects on milk liquidity and the health of the mother and offspring [122]. Experts consistently recommend replacing most forms of *trans* fatty acids with the *cis* forms [122,123]. Food manufacturers have been removing *trans* fats from their formulations for several years and the FDA is considering banning trans fats entirely. Until such time, and due to a history of higher amounts of *trans* oleic acid in the breast milk of American mothers, and lower overall MUFA ingestion in the American diet compared to a European or Mediterranean-style diet, it may be beneficial for the health of pregnant and lactating American mothers and their infants to continue to actively seek out and consume sources of *cis* MUFA—such as those found in oil-containing fruits like avocados and olives.

**Figure 2:**
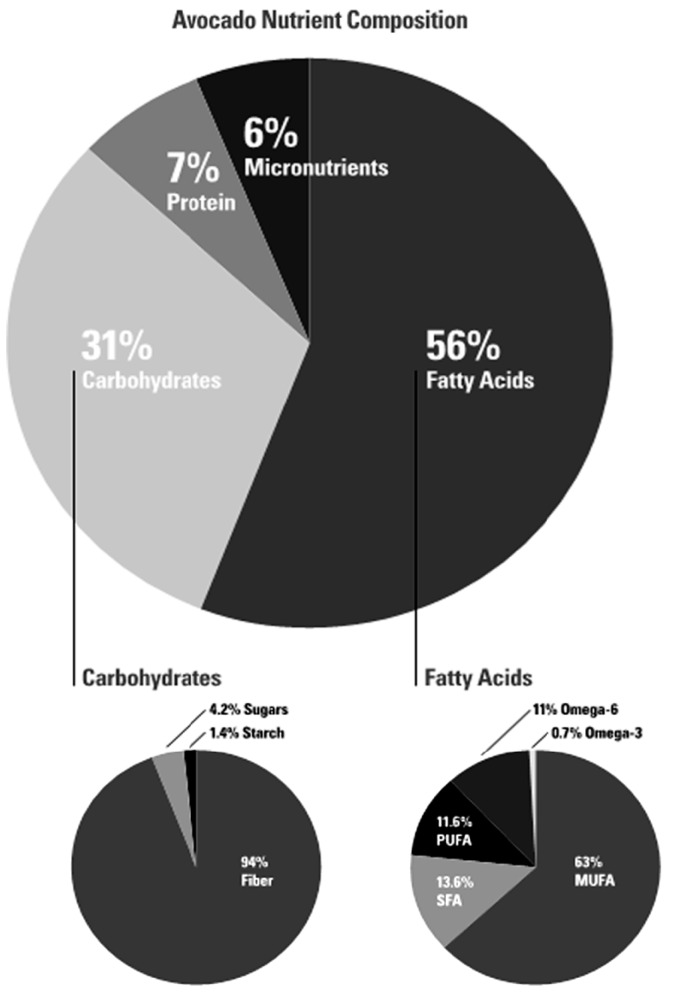
Avocado nutrient composition. Data sourced from: USDA Agricultural Research Service, National Nutrient Database for Standard Reference Release 27. Basic Report: 09038, Avocados, raw, California [7].

According to the Continuing Survey of Food Intakes by Individuals (CSFII), the most common sources of MUFA, not including breast milk, in children ages zero to two years old are cow’s milk, peanut butter, white potato/French fries, hot dogs, eggs, chicken nuggets, corn puffs, and macaroni with cheese [124]. All of these sources, except peanut butter, have higher levels of SFA and/or starchy carbohydrates, than they do MUFA. In comparison, oil-containing fruits such as avocados have much higher levels of MUFA than any other fatty acids, and they also contain more fiber than starch, making them an optimal source of MUFA with likely no nutritional components associated with high cholesterol levels and insulin resistance (Figure 2). Rather, they have multiple nutritional components that research shows are inversely associated with the risk factors involved in the development of cardiovascular disease and metabolic syndrome [124,125,126].

In sum, MUFA in the form of oleic acid are critical to breast milk quality beyond nutritive role because it reduces the melting point of triglycerides, thus providing the proper liquidity required for the breast milk formation [122]. MUFA, along with PUFA, are also necessary for the proper development of the human nervous system [118], and substantial structural and functional brain development in the first year of life [112]. Maternal and infant MUFA intakes are clearly an area of lactation research that deserves more attention. The future research should focus on how maternal nutrition and early infant nutrition can affect health outcomes in later life stages, especially in regards to the targets of obesity, hypertension, and diabetes—all of which can negatively impact pregnant mothers and the health of their offspring.

### 6.2. Carotenoids

Breast milk carotenoids decrease in concentration over the period of lactation. However, lutein is a carotenoid of particular interest because lutein in breast milk rises with maternal intake [119], represents roughly 25% of the carotenoid in breast milk in the first few days of breastfeeding and actually increases to nearly 50% by the end of the first month [127]. In addition to proper infant eye development, improved cognitive function, and various neuroprotective effects [128,129,130], plasma lutein has also been inversely correlated with oxidative DNA damage [79,131]. Lutein is the most abundant carotenoid in avocados [132]; and it is absorbed in greater quantities from avocados relative to other fruits and vegetables with low or no lipid content [96]. There is growing evidence suggesting that maternal antioxidant intake is an important factor in reducing the risk for abnormal pregnancies and birth defects [133]. Therefore, the intake of foods such as avocados—which have the highest recorded lipophilic antioxidant capacity among fruits and vegetables [134]—may also assist fetal and infant health in ways which have yet to be discovered.

## 7. Conclusions

Maternal nutrition plays a crucial role in influencing fetal growth and birth outcomes. Maternal nutrition also influences breast milk composition of some nutrients (*i.e.*, fatty acids and some vitamins). Avocados are a unique nutrient-rich plant-based food that contain many of the critical nutrients for fetal and infant health and development. They fit within the guidelines for a Mediterranean-style diet (*i.e.*, they contain MUFA, fiber, antioxidants, and are low-glycemic), which is known to be beneficial for disease reduction in most populations including pregnant and lactating populations. Based on this review, avocados offer a range of beneficial nutrients that can make a substantial contribution to a nutrient-rich diet when offered as a staple food for the periconceptional period, as well as during pregnancy and lactation. While they are not currently listed on the ChooseMyPlate.gov website as a recommended fruit or vegetable, they do precisely fit the description of a federally recommended food for a pregnant or lactating population. Avocados contain several recommended nutrients for reproductive health such as folate, potassium, carotenoids, and other key compounds for general health such as fiber, MUFA, and antioxidants. They do not contain empty calories from added sugars, saturated fats, or alcohol, and they are also sodium-free. Future research is required to directly study the effects of inclusion of avocados in the diet on maternal health during each of the key periods from pre-conception to the end of breastfeeding, with an emphasis on both the short-term and long-term health of the mother and offspring.

## Figures and Tables

**Table 1:** California avocado composition (USDA 2015).

	1 Serving, 30 g (1 Ounce)	½ Fruit, 68 g (2.27 Ounces)	Per 100 g (3.33 Ounces)	1 Fruit, 136 g (4.53 Ounces)
**Water** (g)	**22**	**49**	**72**	**98**
**Energy** (kcal)	**50**	**114**	**167**	**227**
**Protein** (g)	**0.6**	**1.3**	**2.0**	**2.7**
**Total Lipids** (g)	**4.6**	**10.5**	**15.4**	**21**
Saturated Fat (g)	0.6	1.5	2.1	2.9
Monounsaturated Fat (g)	2.9	6.7	9.8	13.3
Polyunsaturated Fat (g)	0.5	1.2	1.8	2.5
Cholesterol (mg)	0	0	0	0
Stigmasterol (mg)	1.0	1.5	2.0	3
Campesterol (mg)	2.0	3.5	5.0	7
Beta-Sitosterol (mg)	23	51.5	76	103
**Total Carbohydrate** (g)	**2.6**	**5.9**	**8.6**	**11.8**
Insoluble Fiber (g)	1.4	3.2	4.8	6.4
Soluble Fiber (g)	0.6	1.4	2.0	2.8
Sugars (g)	0.1	0.2	0.3	0.4
**Water-Soluble Vitamins**				
Vitamin C (mg)	2.6	6.0	8.8	12
Thiamin (mg)	0	0.1	0.1	0.1
Riboflavin (mg)	0	0.1	0.1	0.2
Niacin (mg)	0.6	1.3	1.9	2.6
Pantothenic acid (mg)	0.4	1.0	1.5	2.0
Vitamin B-6 (mg)	0.1	0.2	0.3	0.4
Folate (μg)	27	60.5	89	121
Choline (mg)	4.3	9.7	14	19.3
Vitamin B-12 (μg)	0	0	0	0
**Fat-Soluble Vitamins and Carotenoids**				
Vitamin A (μg RAE)	2.0	5.0	7.0	10
Carotene, beta (μg)	19	43	63	86
Carotene, alpha (μg)	7	16.5	24	33
Cryptoxanthin, beta (μg)	8	18.5	27	37
Lutein + zeaxanthin (μg)	81	185	271	369
Vitamin E (α-tocopherol) (mg)	0.6	1.3	2.0	2.7
Vitamin D (μg)	0	0	0	0
Vitamin K1 (phylloquinone) (μg)	6.3	14.3	21	28.6
**Minerals**				
Calcium (mg)	4.0	9.0	13	18
Magnesium (mg)	9.0	19.5	29	39
Phosphorus (mg)	16	36.5	54	73
Potassium (mg)	152	345	507	690
Sodium (mg)	2	5.5	8	11
Iron (mg)	0.2	0.4	0.6	0.8
Zinc (mg)	0.2	0.5	0.7	0.9
Copper (mg)	0.1	0.1	0.2	0.2
Manganese (mg)	0.1	0.1	0.1	0.2
Selenium (ug)	0.1	0.3	0.4	0.5

Data sourced from: USDA Agricultural Research Service, National Nutrient Database for Standard Reference Release 27. Basic Report: 09038, Avocados, raw, California [7].

## References

[1] U.S. Department of Health, Human Services and U.S. Department of Agriculture 2015–2020 Dietary Guidelines for Americans. http://www.cnpp.usda.gov/2015-2020-dietary-guidelines-americans.

[2] U.S. Department of Agriculture, U.S. Department of Health and Human Services Scientific Report of the 2015 Dietary Guidelines Advisory Committee—Advisory Report to the Secretary of Health and Human Services and the Secretary of Agriculture. http://health.gov/dietaryguidelines/2015-scientific-report/pdfs/scientific-report-of-the-2015-dietary-guidelines-advisory-committee.pdf.

[3] WHO/FAO (2004). Vitamin and Mineral Requirements in Human Nutrition: Report of a Joint FAO/WHO Expert Consultation.

[4] Otten J.J., Hellwig J.P., Meyers L.D. (2006). Dietary Reference Intakes: The Essential Guide to Nutrient Requirements.

[5] Christian P., Mullany L.C., Hurley K.M., Katz J., Black R.E. (2015). Nutrition and maternal, neonatal, and child health. Semin. Perinatol..

[6] Raiten D.J., Raghavan R., Porter A., Obbagy J.E., Spahn J.M. (2014). Executive summary: Evaluating the evidence base to support the inclusion of infants and children from birth to 24 months of age in the Dietary Guidelines for Americans—“The B-24 Project”. Am. J. Clin. Nutr..

[7] USDA National Nutrient Database for Standard Reference Release 27, Basic Report: 09038, Avocados, Raw, California. http://www.ars.usda.gov/Services/docs.htm?docid=25706.

[8] Chavarro J.E., Rich-Edwards J.W., Rosner B.A., Willett W.C. (2007). Dietary fatty acid intakes and the risk of ovulatory infertility. Am. J. Clin. Nutr..

[9] Champ M., Hoebler C. (2009). Functional food for pregnant, lactating women and in perinatal nutrition: A role for dietary fibres?. Curr. Opin. Clin. Nutr. Metab. Care.

[10] Vanhees K., Vonhogen I.G., van Schooten F.J., Godschalk R.W. (2014). You are what you eat, and so are your children: The impact of micronutrients on the epigenetic programming of offspring. Cell. Mol. Life Sci..

[11] Twigt J.M., Bolhuis M.E., Steegers E.A., Hammiche F., van Inzen W.G., Laven J.S., Steegers-Theunissen R.P. (2012). The preconception diet is associated with the chance of ongoing pregnancy in women undergoing IVF/ICSI treatment. Hum. Reprod..

[12] Abu-Saad K., Fraser D. (2010). Maternal nutrition and birth outcomes. Epidemiol. Rev..

[13] Mason J.B., Saldanha L.S., Martorell R. (2012). The importance of maternal undernutrition for maternal, neonatal, and child health outcomes: An editorial. Food Nutr. Bull..

[14] Ramakrishnan U., Grant F., Goldenberg T., Zongrone A., Martorell R. (2012). Effect of women’s nutrition before and during early pregnancy on maternal and infant outcomes: A systematic review. Paediatr. Perinat. Epidemiol..

[15] Cetin I., Berti C., Calabrese S. (2010). Role of micronutrients in the periconceptional period. Hum. Reprod. Update.

[16] Cox J.T., Phelan S.T. (2009). Prenatal nutrition: Special considerations. Minerva Ginecol..

[17] Fowler J.K., Evers S.E., Campbell M.K. (2012). Inadequate dietary intakes among pregnant women. Can. J. Diet Pract. Res..

[18] Schatzer M., Rust P., Elmadfa I. (2010). Fruit and vegetable intake in Austrian adults: Intake frequency, serving sizes, reasons for and barriers to consumption, and potential for increasing consumption. Public Health Nutr..

[19] De Weerd S., Steegers E.A., Heinen M.M., van den Eertwegh S., Vehof R.M., Steegers-Theunissen R.P. (2003). Preconception nutritional intake and lifestyle factors: First results of an explorative study. Eur. J. Obstet. Gynecol. Reprod. Biol..

[20] Siega-Riz A.M., King J.C., American Dietetic Association, American Society of Nutrition (2009). Position of the American Dietetic Association and American Society for Nutrition: Obesity, reproduction, and pregnancy outcomes. J. Am. Diet Assoc..

[21] Heslehurst N., Ells L.J., Simpson H., Batterham A., Wilkinson J., Summerbell C.D. (2007). Trends in maternal obesity incidence rates, demographic predictors, and health inequalities in 36,821 women over a 15-year period. BJOG.

[22] Ferrara A. (2007). Increasing prevalence of gestational diabetes mellitus: A public health perspective. Diabetes Care.

[23] Parker S.E., Werler M.M., Shaw G.M., Anderka M., Yazdy M.M., National Birth Defects Prevention Study (2012). Dietary Glycemic Index and the risk of birth defects. Am. J. Epidemiol..

[24] Matias S.L., Dewey K.G., Quesenberry C.P., Gunderson E.P. (2014). Maternal prepregnancy obesity and insulin treatment during pregnancy are independently associated with delayed lactogenesis in women with recent gestational diabetes mellitus. Am. J. Clin. Nutr..

[25] ChooseMyPlate.gov Health and Nutrition Information for Pregnant and Breastfeeding Women: Making Healthy Choices in Each Food Group. http://www.choosemyplate.gov/pregnancy-breastfeeding/making-healthy-food-choices.html.

[26] Lundqvist A., Johansson I., Wennberg A., Hultdin J., Hogberg U., Hamberg K., Sandstrom H. (2014). Reported dietary intake in early pregnant compared to non-pregnant women inverted question mark a cross-sectional study. BMC Pregnancy Childbirth.

[27] Picciano M.F., McGuire M.K. (2009). Use of dietary supplements by pregnant and lactating women in North America. Am. J. Clin. Nutr..

[28] ChooseMyPlate.gov Health and Nutrition Information for Pregnant and Breastfeeding Women: Nutritional Needs during Pregnancy. http://www.choosemyplate.gov/pregnancy-breastfeeding/pregnancy-nutritional-needs.html.

[29] Verger E.O., Holmes B.A., Huneau J.F., Mariotti F. (2014). Simple changes within dietary subgroups can rapidly improve the nutrient adequacy of the diet of French adults. J. Nutr..

[30] Kelly T.N., Gu D., Rao D.C., Chen J., Chen J., Cao J., Li J., Lu F., Ma J., Mu J. (2012). Maternal history of hypertension and blood pressure response to potassium intake: The gensalt study. Am. J. Epidemiol..

[31] Qiu C., Coughlin K.B., Frederick I.O., Sorensen T.K., Williams M.A. (2008). Dietary fiber intake in early pregnancy and risk of subsequent preeclampsia. Am. J. Hypertens..

[32] Hernandez T.L., Anderson M.A., Chartier-Logan C., Friedman J.E., Barbour L.A. (2013). Strategies in the nutritional management of gestational diabetes. Clin. Obstet. Gynecol..

[33] Frederick I.O., Williams M.A., Dashow E., Kestin M., Zhang C., Leisenring W.M. (2005). Dietary fiber, potassium, magnesium and calcium in relation to the risk of preeclampsia. J. Reprod. Med..

[34] Centers for Disease Control and Prevention (CDC) Faststats: Infertility. http://www.cdc.gov/nchs/fastats/infertility.htm25706.

[35] Chavarro J., Willett W., Skerrett P.J. (2008). The Fertility Diet: Groundbreaking Research Reveals Natural Ways to Boost Ovulation & Improve Your Chances of Getting Pregnant.

[36] Kulak D., Polotsky A.J. (2013). Should the ketogenic diet be considered for enhancing fertility?. Maturitas.

[37] Crosignani P.G., Vegetti W., Colombo M., Ragni G. (2002). Resumption of fertility with diet in overweight women. Reprod. Biomed. Online.

[38] Santangelo C., Vari R., Scazzocchio B., Filesi C., Masella R. (2014). Management of reproduction and pregnancy complications in maternal obesity: Which role for dietary polyphenols?. Biofactors.

[39] Khoury J., Henriksen T., Christophersen B., Tonstad S. (2005). Effect of a cholesterol-lowering diet on maternal, cord, and neonatal lipids, and pregnancy outcome: A randomized clinical trial. Am. J. Obstet. Gynecol..

[40] Mediterranean Diet Pyramid Poster. http://www.californiaavocado.com/mediterranean-diet/.

[41] Timmermans S., Steegers-Theunissen R.P., Vujkovic M., Bakker R., den Breeijen H., Raat H., Russcher H., Lindemans J., Hofman A., Jaddoe V.W. (2011). Major dietary patterns and blood pressure patterns during pregnancy: The generation R study. Am. J. Obstet. Gynecol..

[42] Toledo E., Lopez-del Burgo C., Ruiz-Zambrana A., Donazar M., Navarro-Blasco I., Martinez-Gonzalez M.A., de Irala J. (2011). Dietary patterns and difficulty conceiving: A nested case-control study. Fertil. Steril..

[43] Hirai S., Takahashi N., Goto T., Lin S., Uemura T., Yu R., Kawada T. (2010). Functional food targeting the regulation of obesity-induced inflammatory responses and pathologies. Mediat. Inflamm..

[44] Sinska B., Kucharska A., Dmoch-Gajzlerska E. (2014). The Diet in improving fertility in women. Pol. Merkur. Lekarski..

[45] Becker G.F., Passos E.P., Moulin C.C. (2015). Short-term effects of a hypocaloric diet with low glycemic index and low glycemic load on body adiposity, metabolic variables, ghrelin, leptin, and pregnancy rate in overweight and obese infertile women: A randomized controlled trial. Am. J. Clin. Nutr..

[46] De Lorgeril M., Salen P., Paillard F., Laporte F., Boucher F., de Leiris J. (2002). Mediterranean diet and the French paradox: Two distinct biogeographic concepts for one consolidated scientific theory on the role of nutrition in coronary heart disease. Cardiovasc. Res..

[47] USDA National Nutrient Database for Standard Reference Release 27: Sugars, Total (G): “Compared to Fruits and Fruit Juices”. https://ndb.nal.usda.gov/.

[48] USDA National Nutrient Database for Standard Reference Release 27: Fiber, Total Dietary (G): “Compared to Fruits and Fruit Juices”. https://ndb.nal.usda.gov/.

[49] Foster-Powell K., Holt S.H., Brand-Miller J.C. (2002). International table of glycemic index and glycemic load values: 2002. Am. J. Clin. Nutr..

[50] Moses R.G., Barker M., Winter M., Petocz P., Brand-Miller J.C. (2009). Can a low-glycemic index diet reduce the need for insulin in gestational diabetes mellitus? A randomized trial. Diabetes Care.

[51] McGowan C.A., Walsh J.M., Byrne J., Curran S., McAuliffe F.M. (2013). The influence of a low glycemic index dietary intervention on maternal dietary intake, glycemic index and gestational weight gain during pregnancy: A randomized controlled trial. Nutr. J..

[52] Kizirian N.V., Kong Y., Muirhead R., Brodie S., Garnett S.P., Petocz P., Sim K.A., Celermajer D.S., Louie J.C., Markovic T.P. (2016). Effects of a low-glycemic index diet during pregnancy on offspring growth, body composition, and vascular health: A pilot randomized controlled trial. Am. J. Clin. Nutr..

[53] Horan M.K., McGowan C.A., Gibney E.R., Byrne J., Donnelly J.M., McAuliffe F.M. (2016). Maternal nutrition and glycaemic index during pregnancy impacts on offspring adiposity at 6 months of age-analysis from the rolo randomised controlled trial. Nutrients.

[54] Danielsen I., Granstrom C., Haldorsson T., Rytter D., Hammer Bech B., Henriksen T.B., Vaag A.A., Olsen S.F. (2013). Dietary glycemic index during pregnancy is associated with biomarkers of the metabolic syndrome in offspring at age 20 years. PLoS ONE.

[55] Hung H.C., Joshipura K.J., Jiang R., Hu F.B., Hunter D., Smith-Warner S.A., Colditz G.A., Rosner B., Spiegelman D., Willett W.C. (2004). Fruit and vegetable intake and risk of major chronic disease. J. Natl. Cancer Inst..

[56] Tobias M., Turley M., Stefanogiannis N., Vander Hoorn S., Lawes C., Mhurchu C.N., Rodgers A. (2006). Vegetable and fruit intake and mortality from chronic disease in New Zealand. Aust. N. Z. J. Public Health.

[57] Liu R.H. (2013). Health-promoting components of fruits and vegetables in the diet. Adv. Nutr..

[58] Murphy M.M., Stettler N., Smith K.M., Reiss R. (2014). Associations of consumption of fruits and vegetables during pregnancy with infant birth weight or small for gestational age births: A systematic review of the literature. Int. J. Womens Health.

[59] Wojdylo A., Oszmianski J. (2009). Bioactive compounds of selected fruit juices. Nat. Prod. Commun..

[60] Stoewsand G.S. (1995). Bioactive organosulfur phytochemicals in Brassica oleracea vegetables—A review. Food Chem. Toxicol..

[61] Grieger J.A., Clifton V.L. (2015). A review of the impact of dietary intakes in human pregnancy on infant birthweight. Nutrients.

[62] Loy S.L., Marhazlina M., Azwany Y.N., Hamid Jan J.M. (2011). Higher intake of fruits and vegetables in pregnancy is associated with birth size. Southeast Asian J. Trop. Med. Public Health.

[63] Procter S.B., Campbell C.G. (2014). Position of the academy of nutrition and dietetics: Nutrition and lifestyle for a healthy pregnancy outcome. J. Acad. Nutr. Diet.

[64] Knudsen V.K., Orozova-Bekkevold I.M., Mikkelsen T.B., Wolff S., Olsen S.F. (2008). Major dietary patterns in pregnancy and fetal growth. Eur. J. Clin. Nutr..

[65] Hanley B., Dijane J., Fewtrell M., Grynberg A., Hummel S., Junien C., Koletzko B., Lewis S., Renz H., Symonds M. (2010). Metabolic imprinting, programming and epigenetics—A review of present priorities and future opportunities. Br. J. Nutr..

[66] Organization W.H. (2006). Promoting Optimal Fetal Development: Report of a Technical Consultation.

[67] Gesteiro E., Rodriguez Bernal B., Bastida S., Sanchez-Muniz F.J. (2012). Maternal diets with low healthy eating index or mediterranean diet adherence scores are associated with high cord-blood insulin levels and insulin resistance markers at birth. Eur. J. Clin. Nutr..

[68] Krapels I.P., van Rooij I.A., Ocke M.C., West C.E., van der Horst C.M., Steegers-Theunissen R.P. (2004). Maternal nutritional status and the risk for orofacial cleft offspring in humans. J. Nutr..

[69] Groenen P.M., van Rooij I.A., Peer P.G., Ocke M.C., Zielhuis G.A., Steegers-Theunissen R.P. (2004). Low maternal dietary intakes of iron, magnesium, and niacin are associated with spina bifida in the offspring. J. Nutr..

[70] Brantsaeter A.L., Haugen M., Samuelsen S.O., Torjusen H., Trogstad L., Alexander J., Magnus P., Meltzer H.M. (2009). A dietary pattern characterized by high intake of vegetables, fruits, and vegetable oils is associated with reduced risk of preeclampsia in nulliparous pregnant norwegian women. J. Nutr..

[71] Ley S.H., Hanley A.J., Retnakaran R., Sermer M., Zinman B., O’Connor D.L. (2011). Effect of macronutrient intake during the second trimester on glucose metabolism later in pregnancy. Am. J. Clin. Nutr..

[72] Rao S., Yajnik C.S., Kanade A., Fall C.H., Margetts B.M., Jackson A.A., Shier R., Joshi S., Rege S., Lubree H. (2001). Intake of micronutrient-rich foods in rural indian mothers is associated with the size of their babies at birth: Pune maternal nutrition study. J. Nutr..

[73] Schisterman E.F., Mumford S.L., Browne R.W., Barr D.B., Chen Z., Louis G.M. (2014). Lipid concentrations and couple fecundity: The life study. J. Clin. Endocrinol. Metab..

[74] Agostoni C., Galli C., Riva E., Rise P., Colombo C., Giovannini M., Marangoni F. (2011). Whole blood fatty acid composition at birth: From the maternal compartment to the infant. Clin. Nutr..

[75] Agostoni C., Marangoni F., Stival G., Gatelli I., Pinto F., Rise P., Giovannini M., Galli C., Riva E. (2008). Whole blood fatty acid composition differs in term versus mildly preterm infants: Small versus matched appropriate for gestational age. Pediatr. Res..

[76] Canovas-Conesa A., Gomariz-Penalver V., Sanchez-Sauco M.F., Jaimes Vega D.C., Ortega-Garcia J.A., Aranda Garcia M.J., Delgado Marin J.L., Trujillo Ascanio A., Lopez Hernandez F., Ruiz Jimenez J.I. (2013). The Association of adherence to a mediterranean diet during early pregnancy and the risk of gastroschisis in the offspring. Cir. Pediatr..

[77] Yamashita D., Shimizu M., Osumi T. (2005). Mechanism for the action of PPARs. Nihon Rinsho.

[78] Chavarro J.E., Colaci D.S., Afeiche M., Gaskins A.J., Wright D., Toth T.L., Hauser R. Dietary Fat Intake and *in-vitro* Fertilization Outcomes: Saturated Fat Intake is Associated with Fewer Metaphase 2 Oocytes. http://humrep.oxfordjournals.org/content/27/suppl_2/ii78.abstract.

[79] Dreher M.L., Davenport A.J. (2013). Hass avocado composition and potential health effects. Crit. Rev. Food Sci. Nutr..

[80] Blumfield M.L., Hure A.J., Macdonald-Wicks L., Smith R., Collins C.E. (2012). Systematic review and meta-analysis of energy and macronutrient intakes during pregnancy in developed countries. Nutr. Rev..

[81] Gibson R.S., Ferguson E.L., Lehrfeld J. (1998). Complementary foods for infant feeding in developing countries: Their nutrient adequacy and improvement. Eur. J. Clin. Nutr..

[82] Quinn M. (2006). Sustained constipation and subsequent reproductive outcomes: Is there a link?. J. Obstet. Gynaecol..

[83] Zhang C., Liu S., Solomon C.G., Hu F.B. (2006). Dietary fiber intake, dietary glycemic load, and the risk for gestational diabetes mellitus. Diabetes Care.

[84] Marlett J.A., Cheung T.F. (1997). Database and quick methods of assessing typical dietary fiber intakes using data for 228 commonly consumed foods. J. Am. Diet Assoc..

[85] U.S. Department of Agriculture, U.S. Department of Health and Human Services (2010). Dietary Guidelines for Americans.

[86] Ionescu-Ittu R., Marelli A.J., Mackie A.S., Pilote L. (2009). Prevalence of severe congenital heart disease after folic acid fortification of grain products: Time trend analysis in Quebec, Canada. BMJ.

[87] Scholl T.O., Hediger M.L., Schall J.I., Khoo C.S., Fischer R.L. (1996). Dietary and serum folate: Their influence on the outcome of pregnancy. Am. J. Clin. Nutr..

[88] USDA National Nutrient Database for Standard Reference Release 27: Folate, Food (µg): “Compared to Fruits and Fruit Juices, and Vegetables and Vegetable Products”. https://ndb.nal.usda.gov/.

[89] Fulgoni V.L., Dreher M., Davenport A.J. (2013). Avocado consumption is associated with better diet quality and nutrient intake, and lower metabolic syndrome risk in US adults: Results from the National Health and Nutrition Examination Survey (NHANES) 2001–2008. Nutr. J..

[90] Azais-Braesco V., Pascal G. (2000). Vitamin a in pregnancy: Requirements and safety limits. Am. J. Clin Nutr..

[91] Elmadfa I., Meyer A.L. (2012). Vitamins for the first 1000 days: Preparing for life. Int. J. Vitam. Nutr. Res..

[92] Ruhl R. (2013). Non-pro-vitamin a and pro-vitamin a carotenoids in atopy development. Int. Arch. Allergy Immunol..

[93] Ruhl R. (2007). Effects of dietary retinoids and carotenoids on immune development. Proc. Nutr. Soc..

[94] Henriksen B.S., Chan G.M. (2014). Importance of carotenoids in optimizing eye and brain development. J. Pediatr. Gastroenterol. Nutr..

[95] Henriksen B.S., Chan G., Hoffman R.O., Sharifzadeh M., Ermakov I.V., Gellermann W., Bernstein P.S. (2013). Interrelationships between maternal carotenoid status and newborn infant macular pigment optical density and carotenoid status. Investig. Ophthalmol. Vis. Sci..

[96] Unlu N.Z., Bohn T., Clinton S.K., Schwartz S.J. (2005). Carotenoid absorption from salad and salsa by humans is enhanced by the addition of avocado or avocado oil. J. Nutr..

[97] Sommerburg O., Keunen J.E., Bird A.C., van Kuijk F.J. (1998). Fruits and vegetables that are sources for lutein and zeaxanthin: The macular pigment in human eyes. Br. J. Ophthalmol..

[98] Lima N.K., Abbasi F., Lamendola C., Reaven G.M. (2009). Prevalence of insulin resistance and related risk factors for cardiovascular disease in patients with essential hypertension. Am. J. Hypertens..

[99] Maillot M., Monsivais P., Drewnowski A. (2013). Food pattern modeling shows that the 2010 dietary guidelines for sodium and potassium cannot be met simultaneously. Nutr. Res..

[100] Seely E.W., Ecker J. (2014). Chronic hypertension in pregnancy. Circulation.

[101] Bateman B.T., Huybrechts K.F., Fischer M.A., Seely E.W., Ecker J.L., Oberg A.S., Franklin J.M., Mogun H., Hernandez-Diaz S. (2015). Chronic hypertension in pregnancy and the risk of congenital malformations: A cohort study. Am. J. Obstet. Gynecol..

[102] Rodrigues S.L., Baldo M.P., Machado R.C., Forechi L., Molina Mdel C., Mill J.G. (2014). High potassium intake blunts the effect of elevated sodium intake on blood pressure levels. J. Am. Soc. Hypertens..

[103] Kazemian E., Dorosty-Motlagh A.R., Sotoudeh G., Eshraghian M.R., Ansary S., Omidian M. (2013). Nutritional status of women with gestational hypertension compared with normal pregnant women. Hypertens. Pregnancy.

[104] Buendia J.R., Bradlee M.L., Daniels S.R., Singer M.R., Moore L.L. (2015). Longitudinal effects of dietary sodium and potassium on blood pressure in adolescent girls. JAMA Pediatr..

[105] USDA National Nutrient Database for Standard Reference Release 27: Potassium, Food (Mg): “Compared to Fruits and Fruit Juices, and Vegetables and Vegetable Products”. https://ndb.nal.usda.gov/.

[106] Rocquelin G., Tapsoba S., Dop M.C., Mbemba F., Traissac P., Martin-Prevel Y. (1998). Lipid content and essential fatty acid (EFA) composition of mature congolese breast milk are influenced by mothers’ nutritional status: Impact on infants’ EFA supply. Eur. J. Clin. Nutr..

[107] Mennella J.A., Beauchamp G.K. (1991). Maternal diet alters the sensory qualities of human milk and the nursling’s behavior. Pediatrics.

[108] Mennella J.A. (2007). The Chemical Senses and the Development of Flavor Preferences in Humans.

[109] Nicklaus S., Boggio V., Chabanet C., Issanchou S. (2005). A prospective study of food variety seeking in childhood, adolescence and early adult life. Appetite.

[110] Cooke L.J., Wardle J., Gibson E.L., Sapochnik M., Sheiham A., Lawson M. (2004). Demographic, familial and trait predictors of fruit and vegetable consumption by pre-school children. Public Health Nutr..

[111] Skinner J.D., Carruth B.R., Bounds W., Ziegler P., Reidy K. (2002). Do food-related experiences in the first 2 years of life predict dietary variety in school-aged children?. J. Nutr. Educ. Behav..

[112] Innis S.M. (2014). Impact of maternal diet on human milk composition and neurological development of infants. Am. J. Clin. Nutr..

[113] Allen L.H. (2012). B vitamins in breast milk: Relative importance of maternal status and intake, and effects on infant status and function. Adv. Nutr..

[114] Innis S.M. (2007). Human milk: Maternal dietary lipids and infant development. Proc. Nutr. Soc..

[115] Jensen R.G. (1996). The lipids in human milk. Prog. Lipid Res..

[116] Da Cunha J., Macedo da Costa T.H., Ito M.K. (2005). Influences of maternal dietary intake and suckling on breast milk lipid and fatty acid composition in low-income women from Brasilia, Brazil. Early Hum. Dev..

[117] Saphier O., Blumenfeld J., Silberstein T., Tzor T., Burg A. (2013). Fatty acid composition of breastmilk of Israeli mothers. Indian Pediatr..

[118] Martysiak-Zurowska D., Zoralska K., Zagierski M., Szlagtys-Sidorkiewicz A. (2011). Fatty acid composition in breast milk of women from Gdansk and the surrounding district in the course of lactation. Med. Wieku Rozwoj..

[119] Del Prado M., Villalpando S., Elizondo A., Rodriguez M., Demmelmair H., Koletzko B. (2001). Contribution of dietary and newly formed arachidonic acid to human milk lipids in women eating a low-fat diet. Am. J. Clin. Nutr..

[120] Insull W., Hirsch J., James T., Ahrens E.H. (1959). The fatty acids of human milk. II. Alterations produced by manipulation of caloric balance and exchange of dietary fats. J. Clin. Investig..

[121] Lopez-Lopez A., Lopez-Sabater M.C., Campoy-Folgoso C., Rivero-Urgell M., Castellote-Bargallo A.I. (2002). Fatty acid and sn-2 fatty acid composition in human milk from Granada (Spain) and in infant formulas. Eur. J. Clin. Nutr..

[122] Jensen R.G. (1999). Lipids in human milk. Lipids.

[123] Ascherio A., Willett W.C. (1997). Health effects of *trans* fatty acids. Am. J. Clin. Nutr..

[124] Nicklas T.A., Hampl J.S., Taylor C.A., Thompson V.J., Heird W.C. (2004). Monounsaturated fatty acid intake by children and adults: Temporal trends and demographic differences. Nutr. Rev..

[125] Root M.M., Dawson H.R. (2013). Dash-like diets high in protein or monounsaturated fats improve metabolic syndrome and calculated vascular risk. Int. J. Vitam. Nutr. Res..

[126] Gillingham L.G., Harris-Janz S., Jones P.J. (2011). Dietary monounsaturated fatty acids are protective against metabolic syndrome and cardiovascular disease risk factors. Lipids.

[127] Cena H., Castellazzi A.M., Pietri A., Roggi C., Turconi G. (2009). Lutein concentration in human milk during early lactation and its relationship with dietary lutein intake. Public Health Nutr..

[128] Johnson E.J. (2014). Role of lutein and zeaxanthin in visual and cognitive function throughout the lifespan. Nutr. Rev..

[129] Ozawa Y., Sasaki M., Takahashi N., Kamoshita M., Miyake S., Tsubota K. (2012). Neuroprotective effects of lutein in the retina. Curr. Pharm. Des..

[130] Vishwanathan R., Kuchan M.J., Sen S., Johnson E.J. (2014). Lutein and preterm infants with decreased concentrations of brain carotenoids. J. Pediatr. Gastroenterol. Nutr..

[131] Haegele A.D., Gillette C., O’Neill C., Wolfe P., Heimendinger J., Sedlacek S., Thompson H.J. (2000). Plasma xanthophyll carotenoids correlate inversely with indices of oxidative dna damage and lipid peroxidation. Cancer Epidemiol. Biomark. Prev..

[132] Ashton O.B., Wong M., McGhie T.K., Vather R., Wang Y., Requejo-Jackman C., Ramankutty P., Woolf A.B. (2006). Pigments in avocado tissue and oil. J. Agric. Food Chem..

[133] Allen L.H. (2005). Multiple micronutrients in pregnancy and lactation: An overview. Am. J. Clin. Nutr..

[134] Wu X., Beecher G.R., Holden J.M., Haytowitz D.B., Gebhardt S.E., Prior R.L. (2004). Lipophilic and hydrophilic antioxidant capacities of common foods in the United States. J. Agric. Food Chem..

